# Supplementary material for: Natural variation in OsMYB8 confers diurnal floret opening time divergence between indica and japonica subspecies
Source: Nat Commun. 2024 Mar 13;15:2262. doi: 10.1038/s41467-024-46579-z (PMC10937712; doi:10.1038/s41467-024-46579-z)
Supplement: Supplementary file 1 — Supplementary Information [file 41467_2024_46579_MOESM1_ESM.pdf]

Natural variation in *OsMYB8* confers diurnal floret opening time divergence between *indica* and *japonica* subspecies

Gou *et al.*

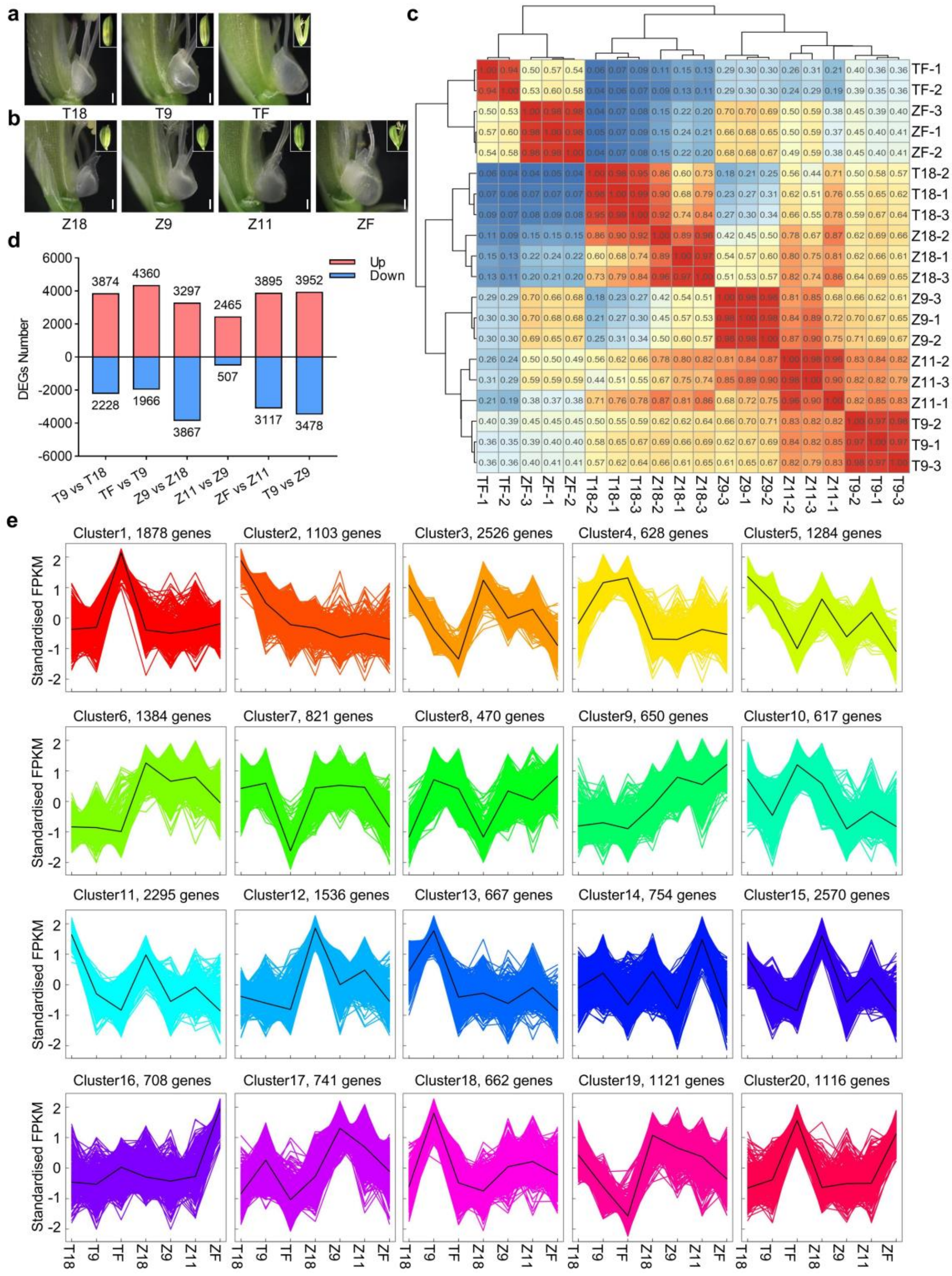

Supplementary Fig. 1. Transcriptomic analysis of lodicules between *indica* and *japonica.* **a, b** Lodicule morphology of TFB (**a**) and ZH11 (**b**) at different time points. T18 and Z18 indicate 18:00 the day before floret opening; T9 and Z9 indicate 9:00 am (1 h and 3 h before floret opening in TFB and ZH11, respectively); Z11 indicate 11:00 am (1 h before opening in ZH11); TF and ZF indicate the time undergoing peak floret opening time (~10:00 am in TFB; ~12:00 noon in ZH11). Scale bars, 250 μm. The

boxed areas indicate the corresponding florets. **c** Correlation heatmap between the biological replicates used for RNA-seq. **d** Number of up-regulated and down-regulated DEGs of the six comparisons (*P*-value < 0.05, absolute $\log_2FC \geq 1$). **e** k-means clustering of normalized gene expression patterns in lodicules of TFB and ZH11 at different time points. Each line depicts the expression pattern of one gene, and the core values for each cluster are plotted in black.

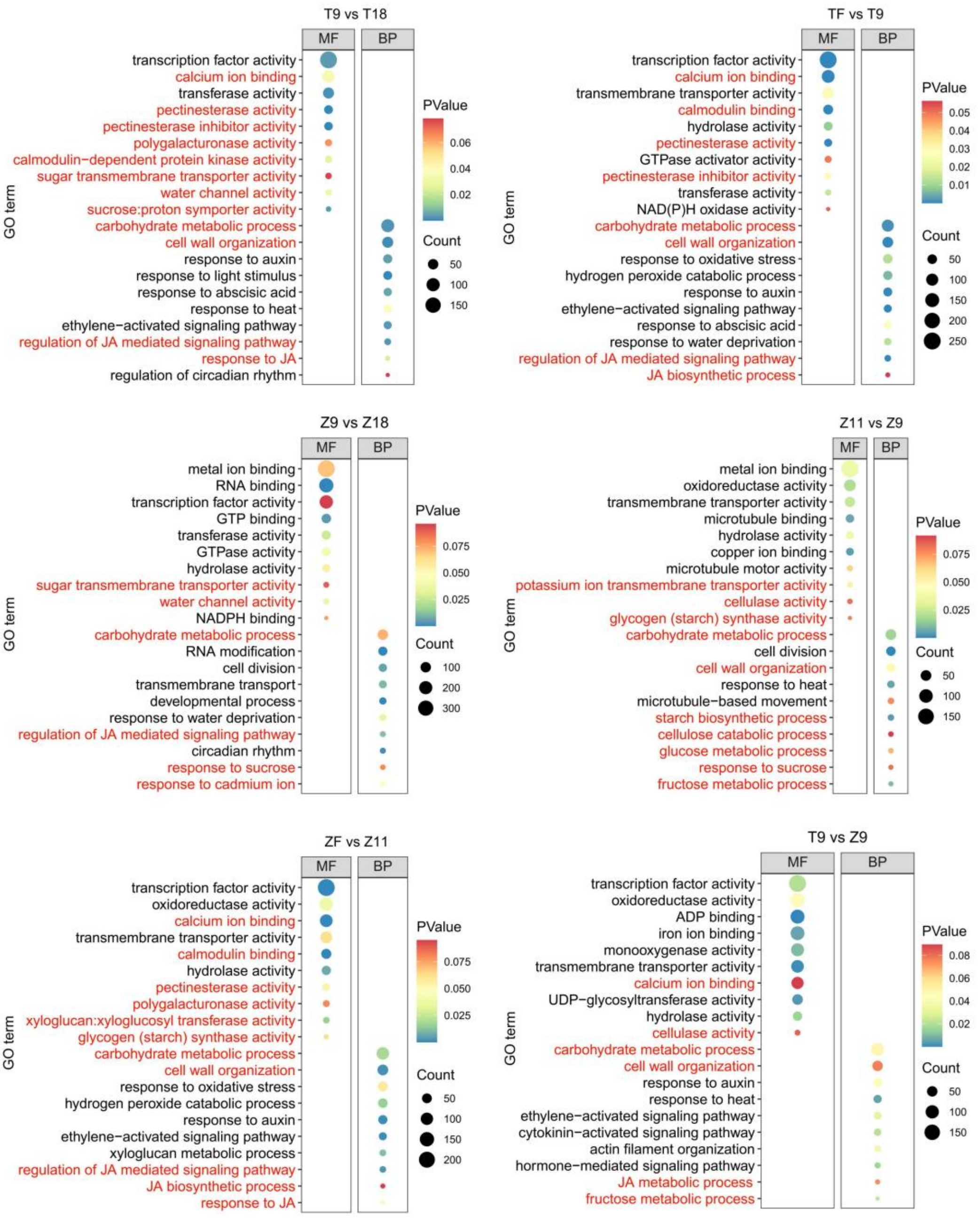

Supplementary Fig. 2. GO enrichment about genes in different comparative group. The biological processes and molecular pathways related JA pathway, cell osmolality, cell wall remodeling and water absorption are highlighted in red. MF indicates molecular function, BP indicates biological process.

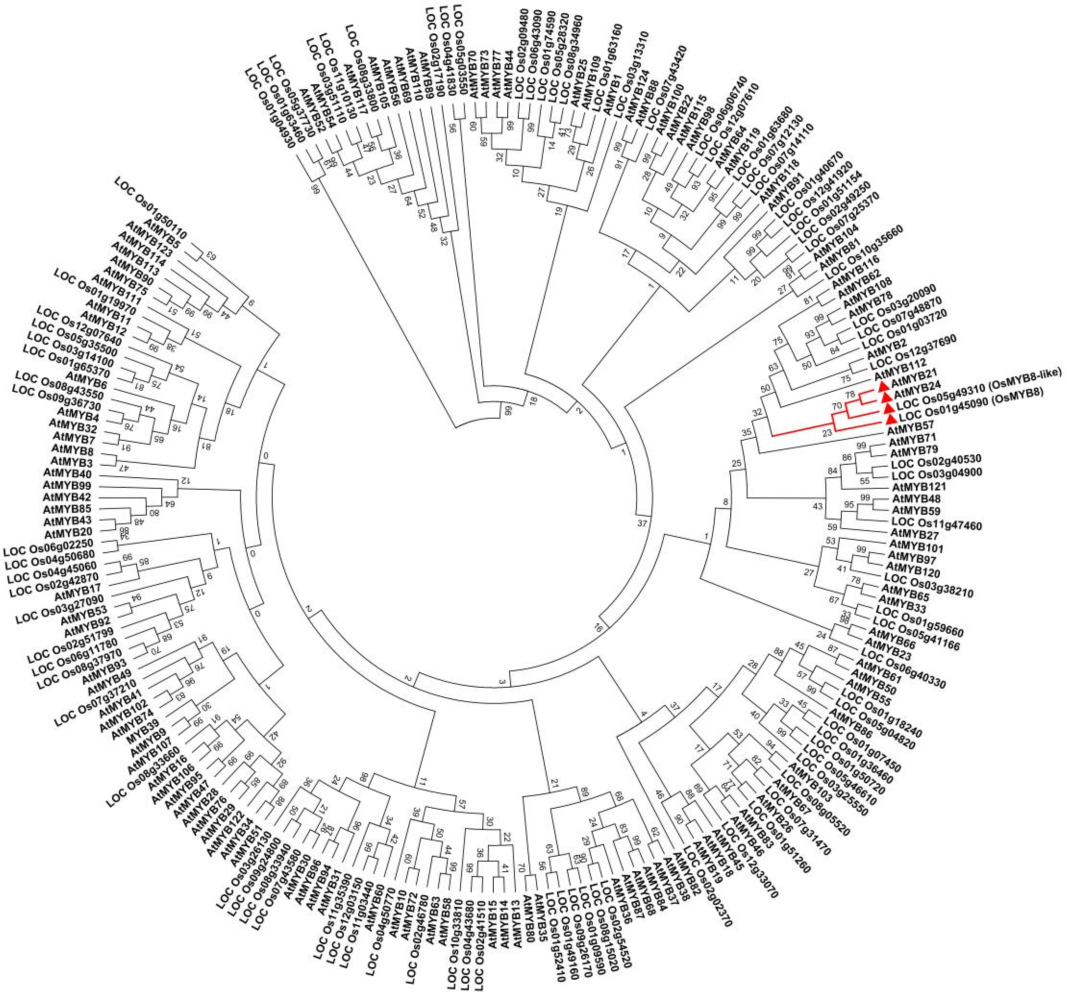

Supplementary Fig. 3. Evolutionary tree of R2R3-MYB transcription factor families in rice and *Arabidopsis*. The unrooted neighbor-joining phylogenetic tree was constructed with MEGA7, based on the full-length amino acid sequences. Numbers above the branches indicate the percentage of bootstrap values calculated from 1,000 replicates. The clade including *OsMYB8* (*LOC_Os01g45090*) and its homologs (*OsMYB8-like*) in rice and in *Arabidopsis* (*AtMYB21/24*) is highlighted in red.

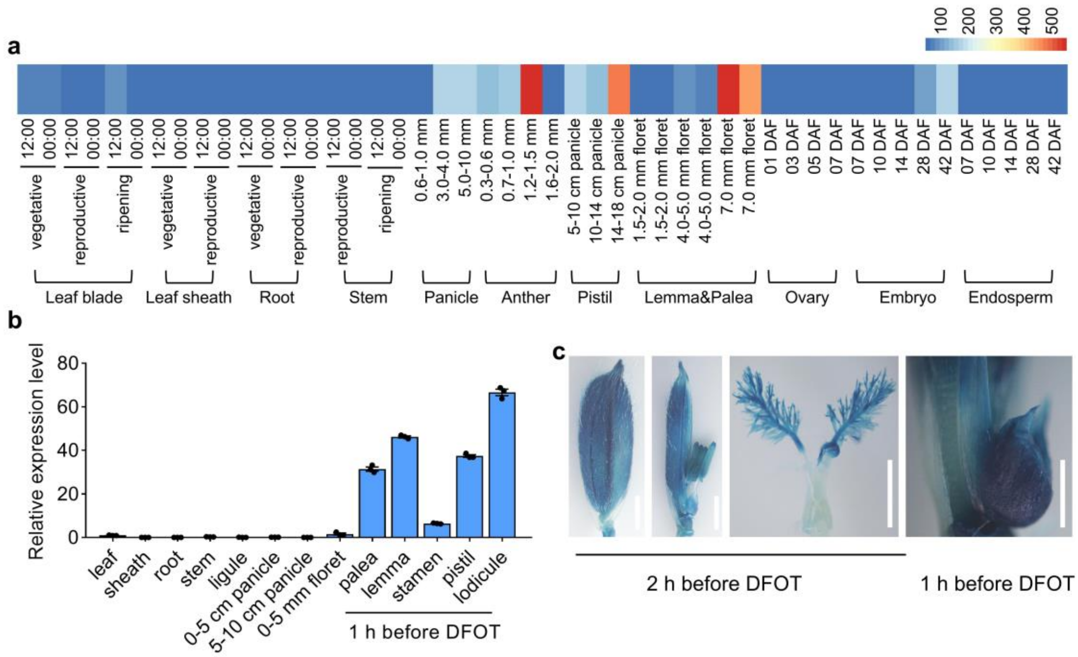

Supplementary Fig. 4. Expression pattern analysis of *OsMYB8*. a Heatmap of *OsMYB8* expression at different developmental stages in a *japonica* cultivar Nipponbare. Data were obtained from a public database (https://ricexpro.dna.affrc.go.jp/). **b** Relative expression levels of *OsMYB8* in various tissues of ZH11. Values are mean ± SEM. (n = 3 biological replicates). **c** GUS-staining analysis of *pOsMYB8::GUS* floret showing strong blue signal in the glume, stamens, stigma and lodicule. Scale bars, 1 mm. Source data are provided as a Source Data file.

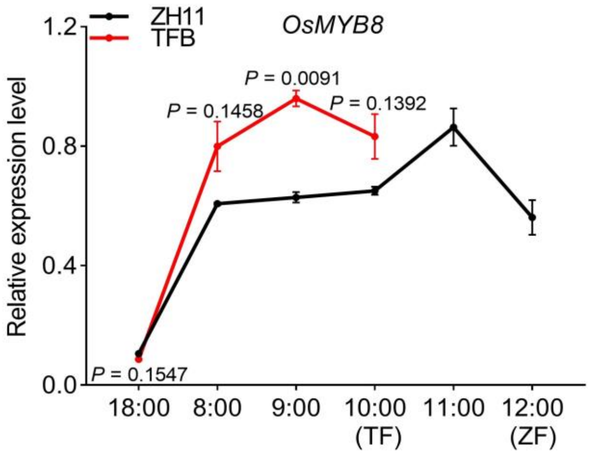

Supplementary Fig. 5. The expression level of *OsMYB8* in the lodicules of TFB and ZH11 at different time points. RT-qPCR showing the relative expression level of *OsMYB8* in the lodicules of TFB and ZH11 at 18:00 the day before floret opening and a series of different time points on the day of floret opening, including 8:00 am, 9:00 am, 10:00 am (peak floret opening time for TFB, TF), 11:00 am and 12:00 noon (peak floret opening time for ZH11, ZF). Values are mean ± SEM. (n = 2 biological replicates). Significance is determined by two-sided Student's *t*-test, and *P* values are indicated. Source data are provided as a Source Data file.

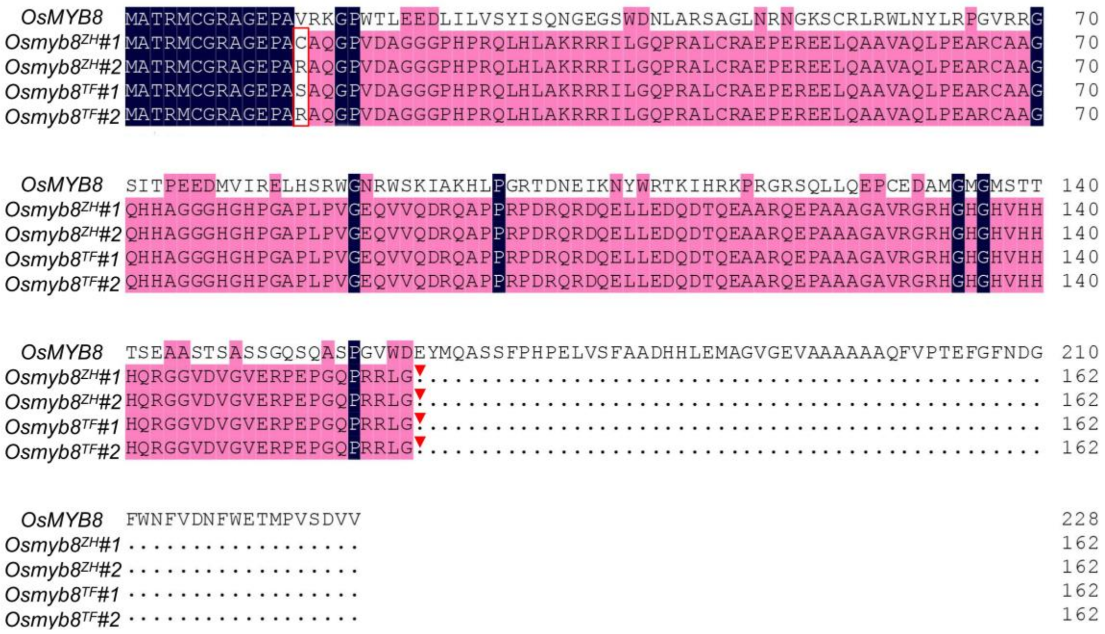

Supplementary Fig. 6. Alignment of the amino acid sequences of *OsMYB8* in WT and the *Osmyb8* mutants. The predictive amino acid sequences of OsMYB8 in the *Osmyb8*^{ZH}*#1*, *Osmyb8*^{ZH}*#2*, *Osmyb8*^{TF}*#1* and *Osmyb8*^{TF}*#2* mutant lines are deduced from their DNA sequencing results. Frame shifts in the *Osmyb8* mutants are indicated by the red box. Red triangles indicate the premature terminations in the *Osmyb8* mutants.

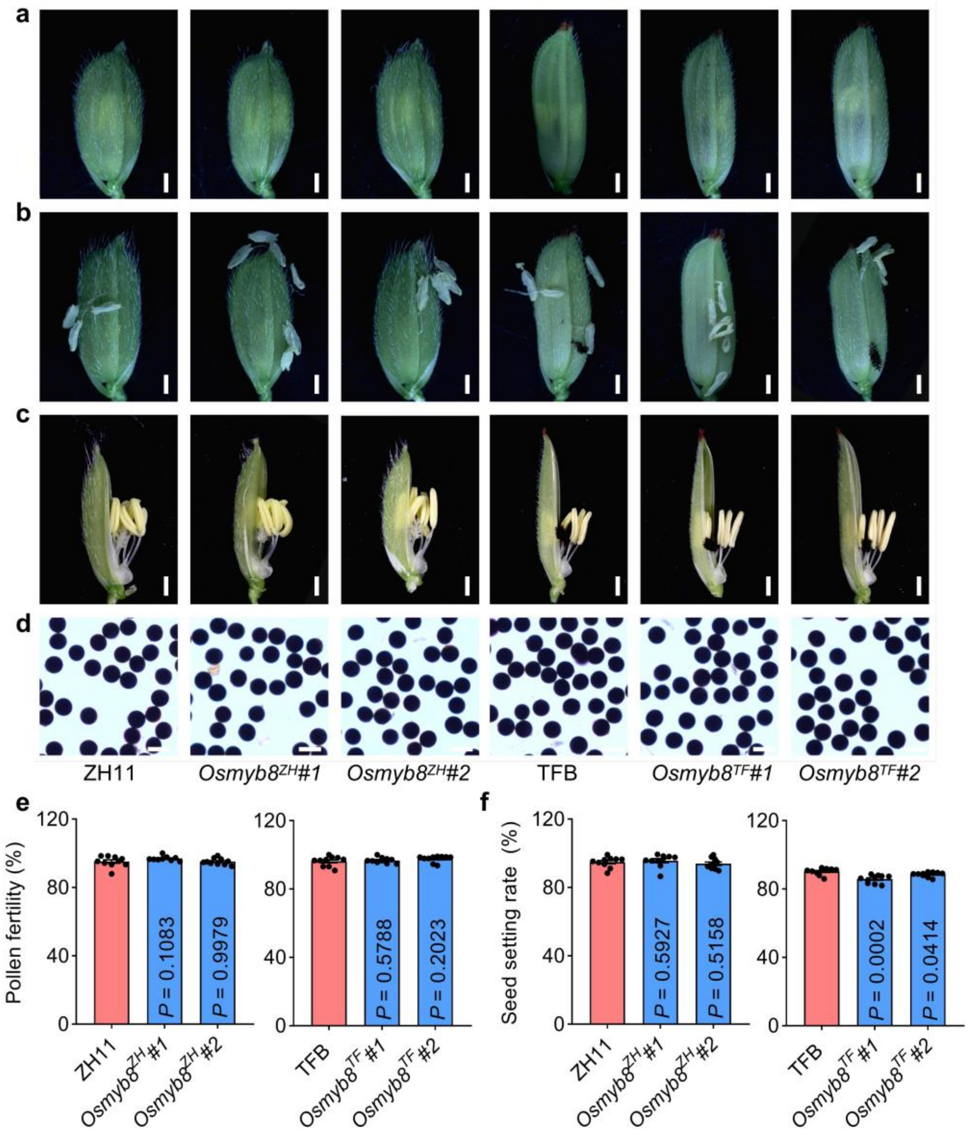

Supplementary Fig. 7. *OsMYB8* did not affect rice fertility and anther dehiscence. **a, b** Florets of ZH11, *Osmyb8*^{ZH}, TFB and *Osmyb8*^{TF} before opening (**a**) and after opening (**b**). Scale bars, 1 mm. **c** Stamens of ZH11, *Osmyb8*^{ZH}, TFB and *Osmyb8*^{TF} before opening. Scale bars, 1 mm. **d** Images of pollens of ZH11, *Osmyb8*^{ZH}, TFB and *Osmyb8*^{TF} stained with 1% I_2-KI (potassium iodide) solution. Scale bars, 50 µm. **e** Pollen fertility of ZH11 and *Osmyb8*^{ZH}, TFB and *Osmyb8*^{TF} based on I_2-KI staining. (n = 10 florets). **f** Seed setting rates of ZH11 and *Osmyb8*^{ZH}, TFB and *Osmyb8*^{TF}. (n = 10 panicles). The values in **e** and **f** are means ± SEM. Significance is evaluated by the two-sided Student's *t*-test, and *P* values are indicated. Source data are provided as a Source Data file.

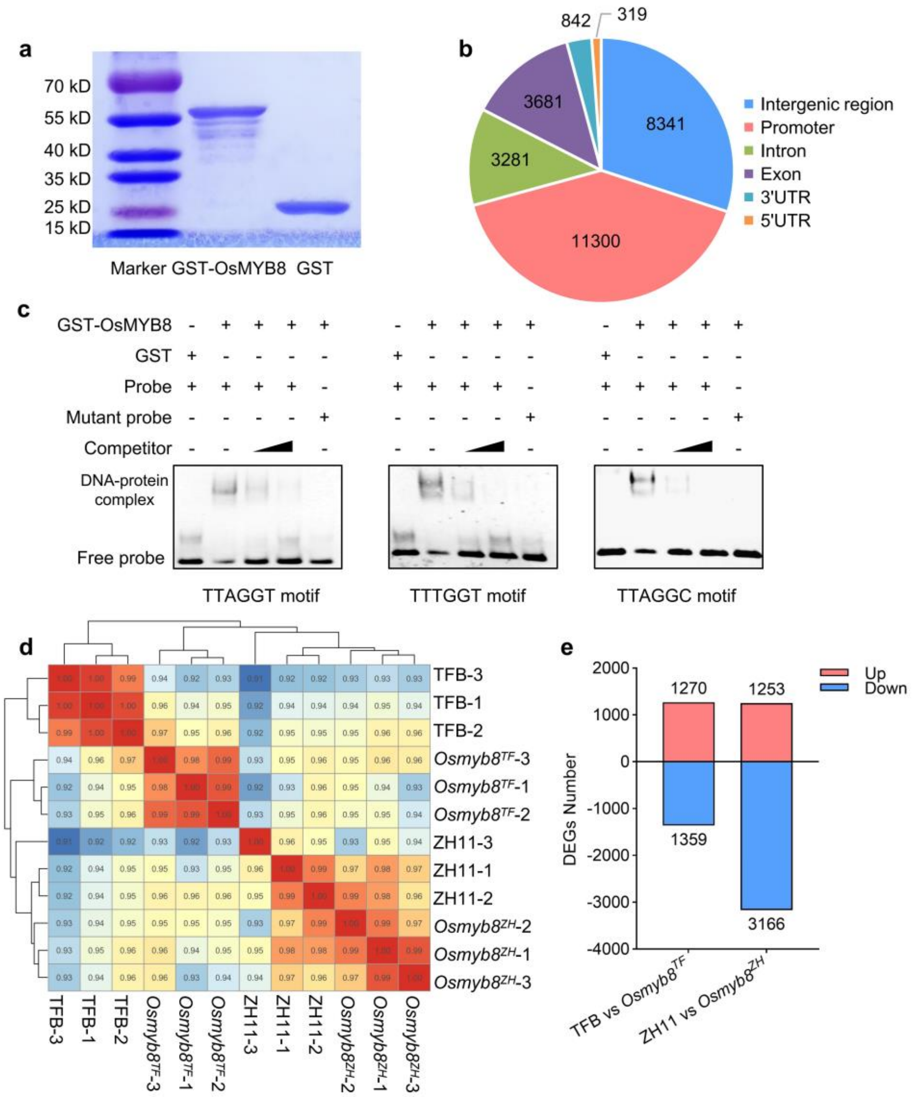

Supplementary Fig. 8. Identification of the genome-wide direct targets of OsMYB8 by DAP-seq and RNA-seq analyses. a Coomassie stained SDS-PAGE gels showing the purity of recombinant GST protein and GST-OsMYB8 fusion protein used for DAP-seq. **b** The number of bound genes by OsMYB8 obtained by DAP-seq with $P < 0.01$ and their distribution in the whole genome. The promoter regions were determined as the binding peaks within 3-kp upstream of ATG. **c** EMSA assays showing that GST-OsMYB8 recombinant protein directly binds to the TTAGGT, TTTGGT and TTAGGC motifs, respectively. Unlabeled probes were used as competitors. GST was used as a negative control. **d** Correlation heatmap of all expressed genes between samples. **e** Number of up-regulated and down-regulated DEGs (P-value < 0.05, absolute $\log_2$FC ≥ 1). Source data are provided as a Source Data file.

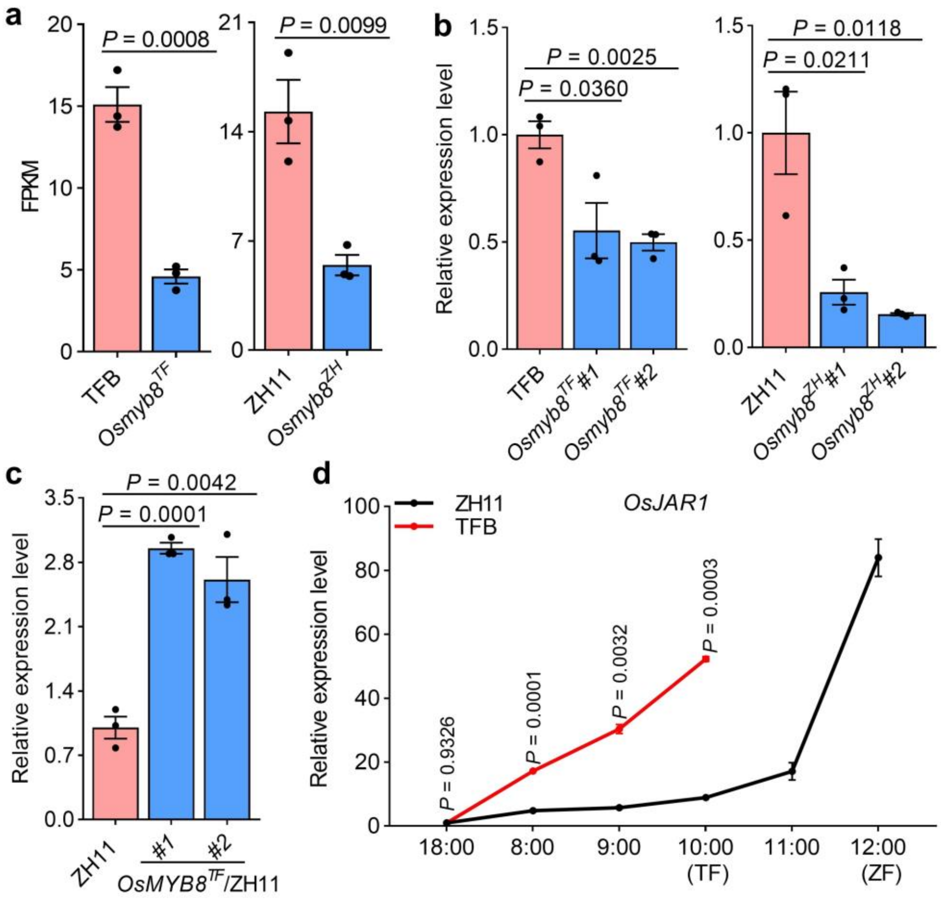

Supplementary Fig. 9. OsMYB8 promote the expression of *OsJAR1*. a The FPKM of *OsJAR1* in the *Osmyb8*^{TF} and *Osmyb8*^{ZH} lodicule transcriptome. (n = 3 biological replicates). **b, c** RT-qPCR analysis of relative expression level of *OsJAR1* in lodicules of the *Osmyb8* mutants (**b**) and *OsMYB8*^{TF}/ZH11 lines (**c**). (n = 3 biological replicates). **d** RT-qPCR analysis of relative expression level of *OsJAR1* in the lodicules of TFB and ZH11 at 18:00 the day before floret opening and different time points on the day of floret opening, including 8:00 am, 9:00 am, 10:00 am (peak floret opening time for TFB, TF), 11:00 am and 12:00 noon (peak floret opening time for ZH11, ZF). (n = 2 biological replicates). The Values in **a-d** are mean ± SEM. Significance is determined by two-sided Student's *t*-test, and *P* values are indicated. Source data are provided as a Source Data file.

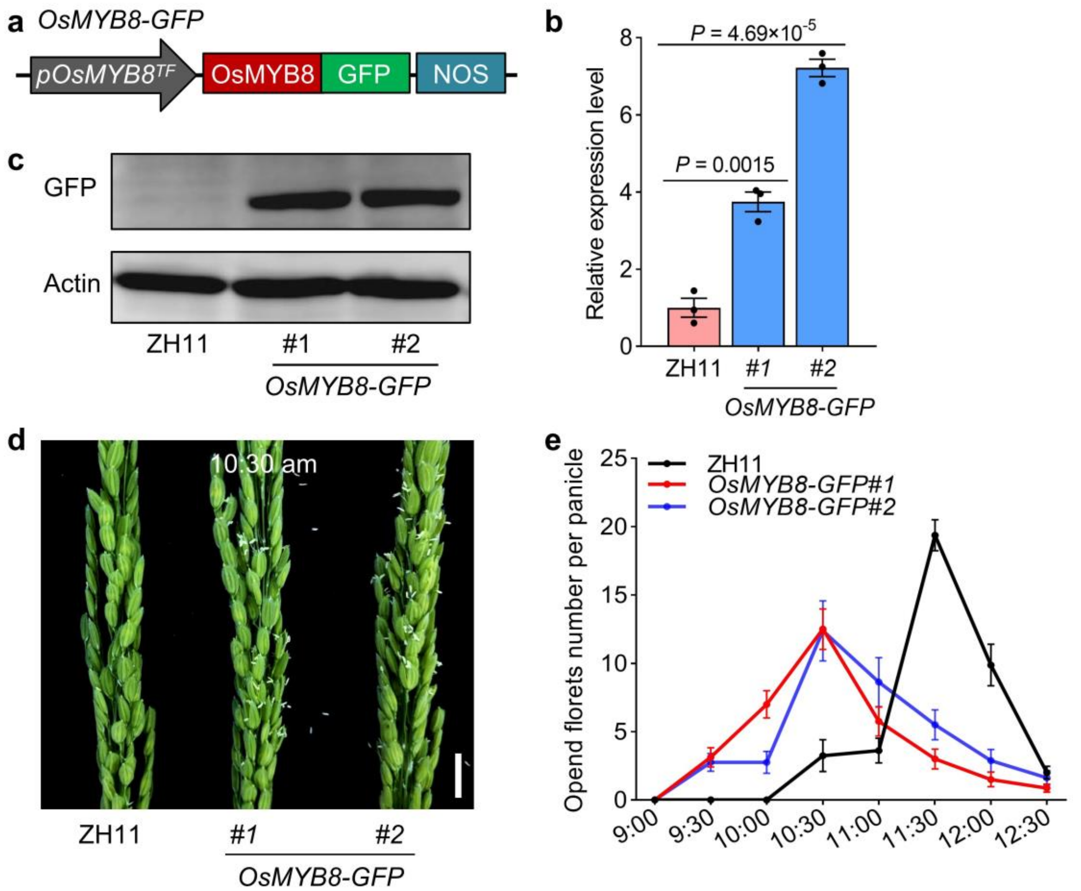

Supplementary Fig. 10. Generation and verification of the *OsMYB8-GFP* transgenic plants. **a** Schematic diagram of the vector used for constructing the *OsMYB8-GFP* materials. *pOsMYB8^{TF}* means that the promoter was amplified from TFB. **b** Relative expression level of *OsMYB8* in the lodicules of ZH11 and *OsMYB8-GFP* transgenic plants. Values are mean ± SEM. (n = 3 biological replicates). Significance is evaluated by the two-sided Student's *t*-test, and *P* values are indicated. **c** Detection of OsMYB8-GFP by immunoblotting with anti-GFP antibody. ZH11 was included as a negative control, and anti-Actin was included for immunoblotting to show similar loadings. Total protein was extracted from florets of ZH11 and the *OsMYB8-GFP* homozygous plants at 1-3 h before opening. **d** Comparison of panicles in ZH11 and the *OsMYB8-GFP* transgenic line at 10:30 am in June 2022 in Guangzhou. Scale bars, 1 cm. **e** Number of opened florets per panicle in ZH11 and the *OsMYB8-GFP* transgenic line at different time points of the day in June 2022 in Guangzhou. Values are means ± SEM. (n = 8 panicles). Source data are provided as a Source Data file.

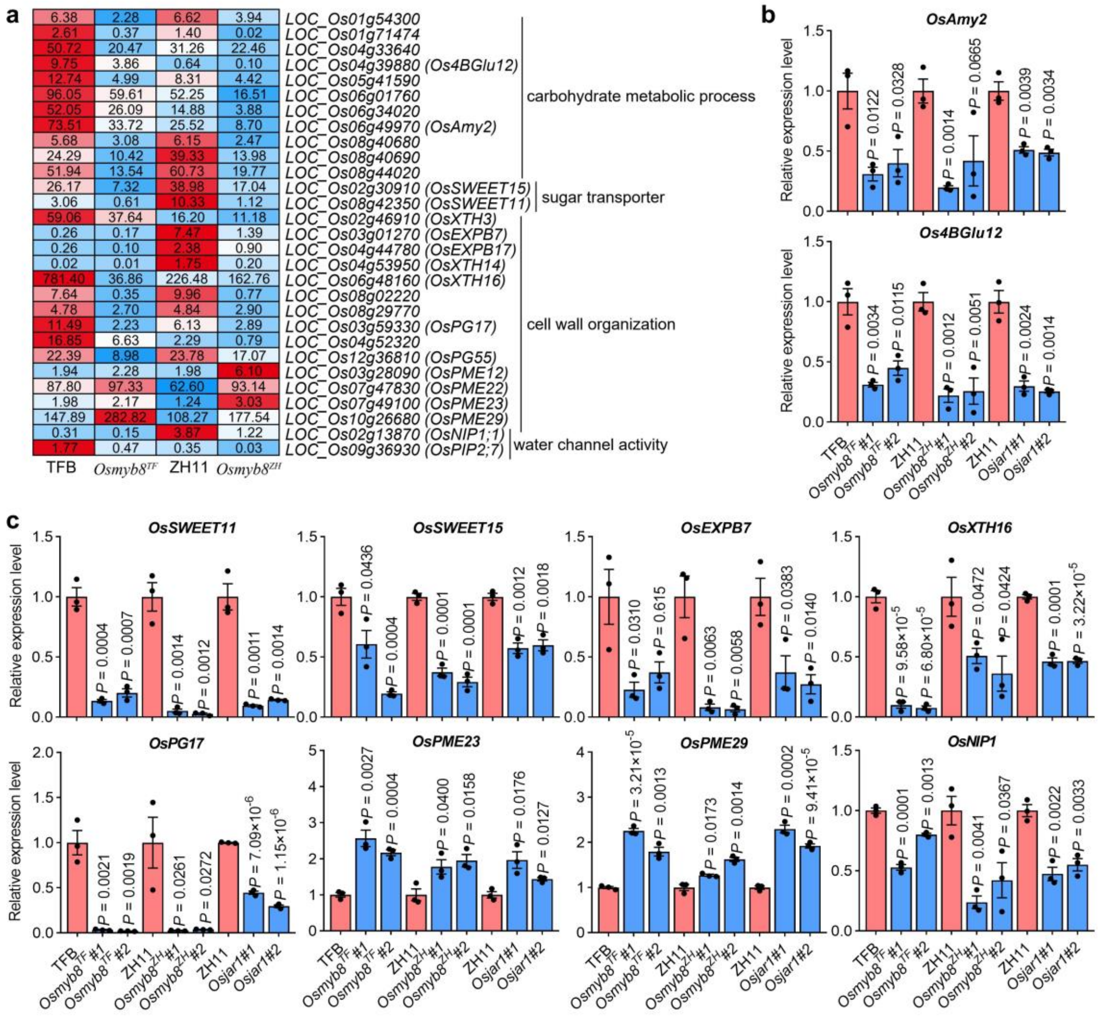

Supplementary Fig. 11. Expression levels of genes related to lodicule hydration and expansion in the *Osmyb8* and *Osjar1* mutants. a A heatmap shows the expression of several genes related to carbohydrate metabolic process, sugar transporters, cell wall organization and water channel activity in the transcriptome of *Osmyb8* lodicules. The numbers in the heatmap represent the average FPKM values. **b, c** RT-qPCR analysis of expression of 10 genes in the lodicules of the *Osmyb8* and *Osjar1* mutants. Values are mean ± SEM. (n = 3 biological replicates). Significance is evaluated by the two-sided Student's *t*-test, and *P* values are indicated. Source data are provided as a Source Data file.

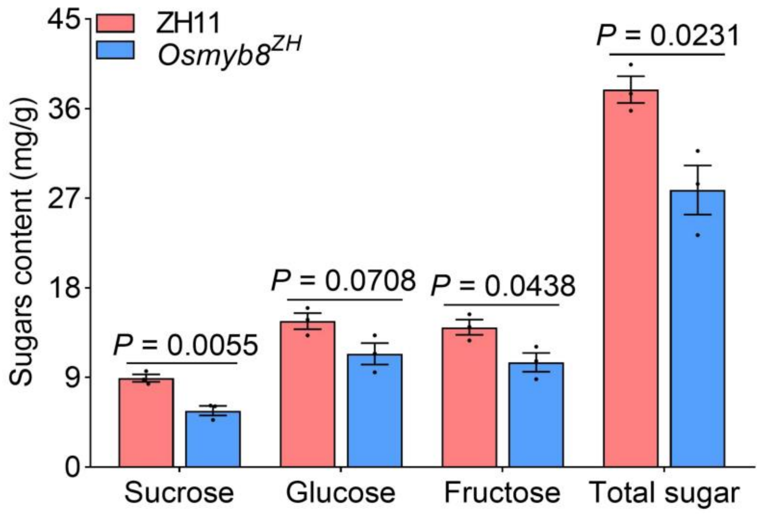

Supplementary Fig. 12. Determination of soluble sugar contents in the lodicules of ZH11 and *Osmyb8*^{ZH}. The sucrose, glucose, fructose and total sugar contents in the lodicules of ZH11 and *Osmyb8*^{ZH} at 10:00 am. Values are mean ± SEM. (n = 3 biological replicates). Significance is evaluated by the two-sided Student's *t*-test, and *P* values are indicated. Source data are provided as a Source Data file.

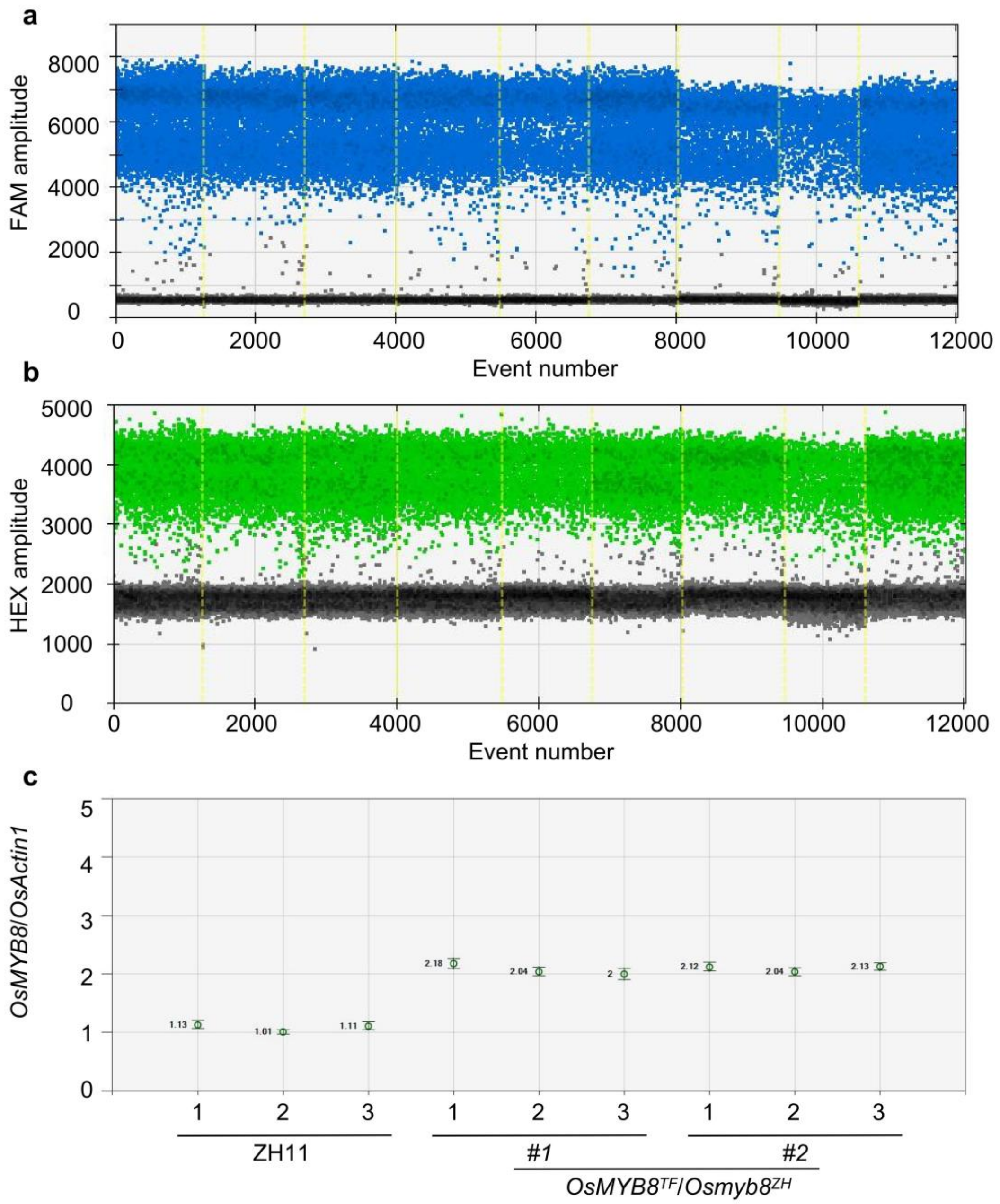

Supplementary Fig. 13. Measurement of transgene copy number of *OsMYB8* using digital droplet PCR (ddPCR). a, b Fluorescence amplitude plots of droplets for *OsMYB8* labeled with the FAM probe (**a**) and for *OsActin1* labeled with the HEX probe (**b**). Each dot represents a single droplet. The horizontal axis (event number) indicates the number of droplets measured across the total experiment and the vertical axis represents the fluorescence amplitude in the FAM channel (**a**) and the HEX channel (**b**). Under each channel, positive droplets with high fluorescence intensity (shown as blue or green dots), and negative droplets with low fluorescence intensity (shown as gray dots). **c** Display of the calculated *OsMYB8* gene copy number values of ZH11 and the

OsMYB8^{TF}/*Osmyb8*^{ZH} transgene plants. *OsActin1* is a single copy gene in the rice genome and was used as an endogenous control in this analysis. The calculation of *OsMYB8* copy number relies on the ratio of positive to negative droplets. Three independent biological replicates were performed for each line. The error bars represent the maximum and minimum poisson distribution for the 95% confidence interval generated by the QuantaSoft™ software. Source data are provided as a Source Data file.

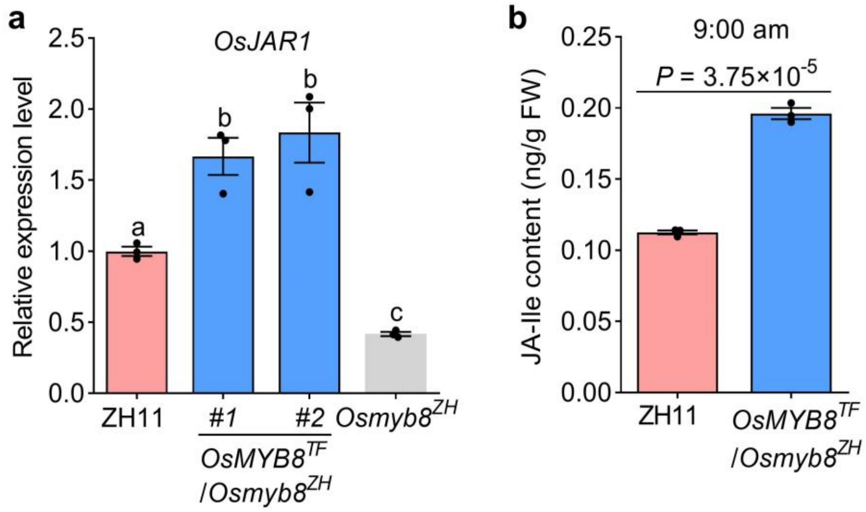

Supplementary Fig. 14. *OsMYB8^TF* elevates *OsJAR1* expression level and JA-Ile content in the *Osmyb8^ZH* mutant. a Relative expression level of *OsJAR1* in lodicules of ZH11, *OsMYB8^TF*/*Osmyb8^ZH* and *Osmyb8^ZH* mutant. Values are mean ± SEM. (n = 3 biological replicates). Letters above the bars indicate significant differences ($P < 0.05$), as evaluated by one-way ANOVA with Tukey's multiple comparisons test. **b** JA-Ile content in lodicules of ZH11 and *OsMYB8^TF*/*Osmyb8^ZH* at 9:00 am. Values are mean ± SEM. (n = 3 biological replicates). Significance is evaluated by the two-sided Student's *t*-test, and *P* values are indicated. Source data are provided as a Source Data file.

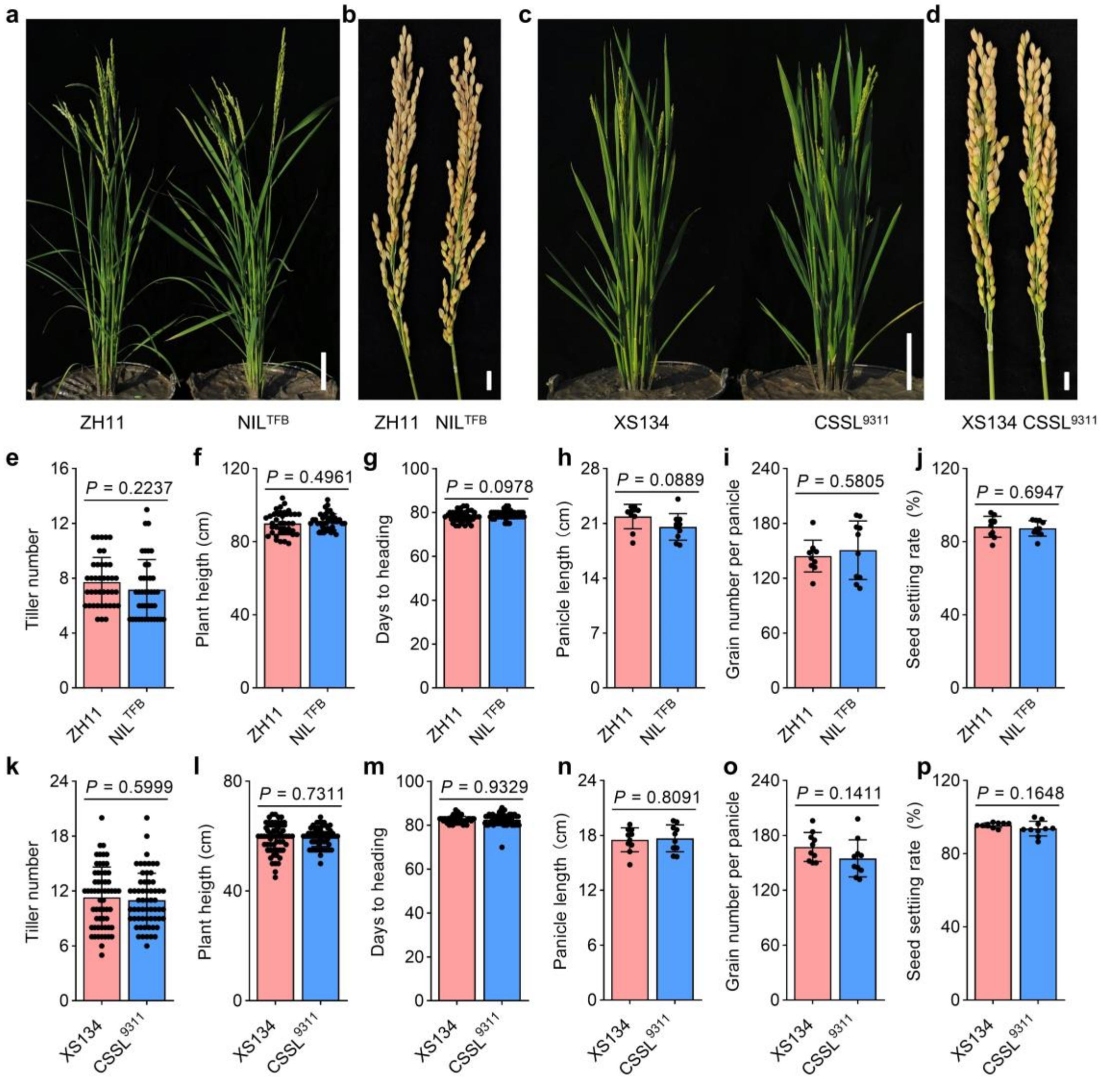

Supplementary Fig. 15. The plant architecture of *OsMYB8*^{Hap1}-carrying NILs. a,c Plant architecture of ZH11 and NIL^{TFB} (**a**) XS134 and CSSL^{9311} (**c**). Scale bars, 10 cm. **b,d** Panicle architecture of ZH11 and NIL^{TFB} (**c**) XS134 and CSSL^{9311} (**d**). Scale bars, 1 cm. **e-g** Tiller number (**e**), plant height **(f)** and heading date (**g**) of ZH11 and NIL^{TFB}. (n = 40 plants). **h-j** Panicle length (**h**), grain number per panicle (**i**) and seed setting rate (**j**) of ZH11 and NIL^{TFB}. (n = 10 panicles). **k-m** Tiller number (**k**), plant height (**l**) and heading date (**m**) of XS134 and CSSL^{9311}. (n = 60 plants). **n-p** Panicle length (**n**), grain number per panicle (**o**) and setting percentage (**p**) of XS134 and CSSL^{9311}. (n = 10 panicles). The values in **e-p** are mean ± SEM. Significance is evaluated by the two-sided Student's *t*-test, and *P* values are indicated. Source data are provided as a Source Data file.

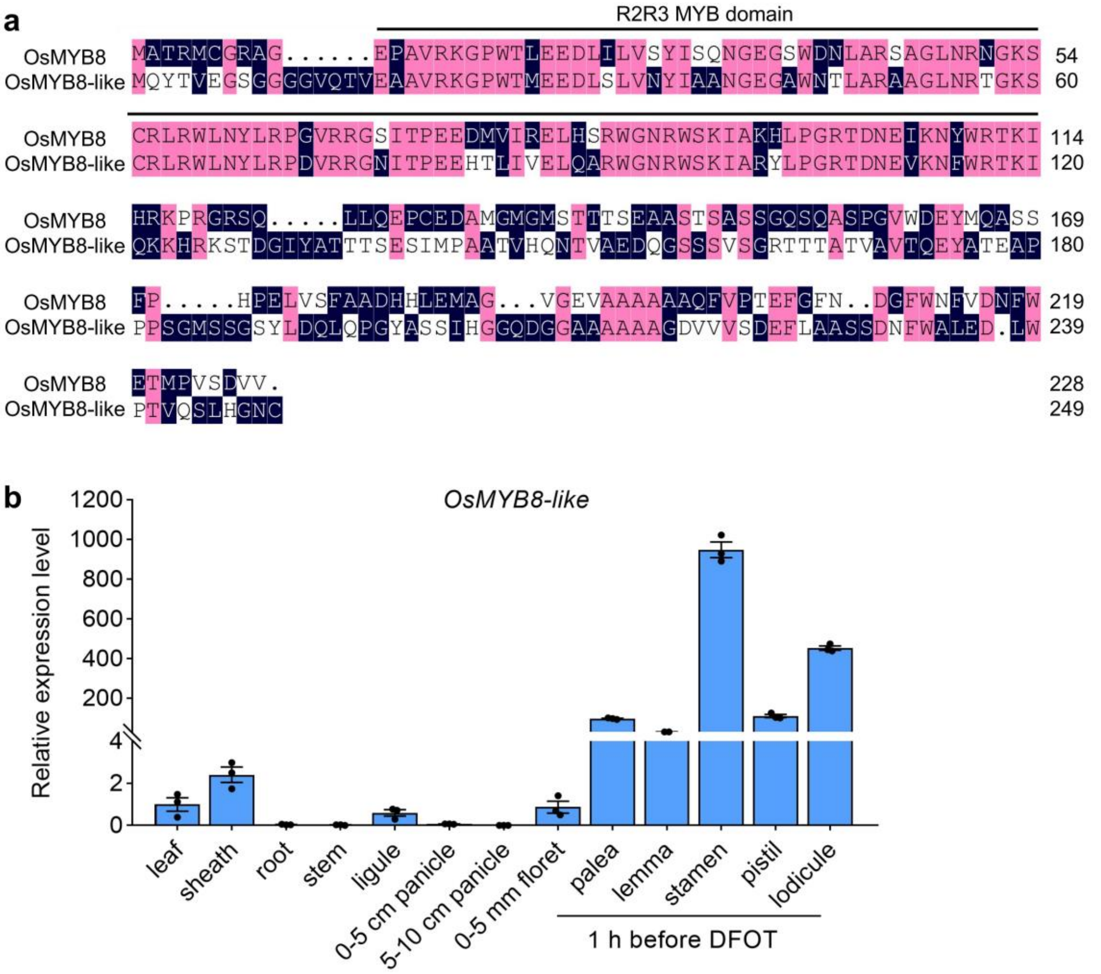

Supplementary Fig. 16. Expression pattern analysis of *OsMYB8-like.* **a** Alignment of the amino acid sequences of OsMYB8 and OsMYB8-like. **b** Relative expression levels of *OsMYB8-like* in various tissues of ZH11. Values are mean ± SEM. (n = 3 biological replicates). Source data are provided as a Source Data file.
