# Supplementary material for: Natural variation in OsMYB8 confers diurnal floret opening time divergence between indica and japonica subspecies
Source: Nat Commun. 2024 Mar 13;15:2262. doi: 10.1038/s41467-024-46579-z (PMC10937712; doi:10.1038/s41467-024-46579-z)
Supplement: Supplementary file 3 — Description of Additional Supplementary Files [file 41467_2024_46579_MOESM3_ESM.pdf]

Description of Additional Supplementary Files

File Name: Supplementary Data 1
Description: The *indica* and *japonica* rice used for DFOT investigation.

File Name: Supplementary Data 2
Description: The differentially expressed genes (DEGs) in the lodicules of TFB and ZH11 at different time points.

File Name: Supplementary Data 3
Description: Identification of OsMYB8-bound genes by DAP-seq.

File Name: Supplementary Data 4
Description: The FPKM of all DEGs in the lodicules of TFB and *Osmyb8*^{TF}.

File Name: Supplementary Data 5
Description: The FPKM of all DEGs in the lodicules of TFB and *Osmyb8*^{ZH}.

File Name: Supplementary Data 6
Description: The list of genes in cell wall modification and JA pathway.

File Name: Supplementary Data 7
Description: Detailed information of the cultivated rice used for haplotype analysis of the *OsMYB8* promoter.

File Name: Supplementary Data 8
Description: Detailed information of the wild rice and cultivated rice used for nucleotide diversity analysis.

File Name: Supplementary Data 9
Description: The nucleotide diversity (π) analysis with 20-kp windows and 2-kb steps throughout Chr1.

File Name: Supplementary Data 10
Description: The weighted F_{ST} analysis with 20-kp windows and 2-kb steps throughout Chr1.

File Name: Supplementary Data 11
Description: Primers used in this study.
