# Supplementary material for: Natural variation in OsMYB8 confers diurnal floret opening time divergence between indica and japonica subspecies
Source: Nat Commun. 2024 Mar 13;15:2262. doi: 10.1038/s41467-024-46579-z (PMC10937712; doi:10.1038/s41467-024-46579-z)
Supplement: Supplementary file 15 — Reporting Summary [file 41467_2024_46579_MOESM15_ESM.pdf]

nature portfolio

Corresponding author(s): Haiyang Wang

Last updated by author(s): Feb 17, 2024

Reporting Summary

Nature Portfolio wishes to improve the reproducibility of the work that we publish. This form provides structure for consistency and transparency in reporting. For further information on Nature Portfolio policies, see our Editorial Policies and the Editorial Policy Checklist.

Statistics

For all statistical analyses, confirm that the following items are present in the figure legend, table legend, main text, or Methods section.

n/a	Confirmed	
☐	☒	The exact sample size (*n*) for each experimental group/condition, given as a discrete number and unit of measurement
☐	☒	A statement on whether measurements were taken from distinct samples or whether the same sample was measured repeatedly
☐	☒	The statistical test(s) used AND whether they are one- or two-sided<br>*Only common tests should be described solely by name; describe more complex techniques in the Methods section.*
☒	☐	A description of all covariates tested
☒	☐	A description of any assumptions or corrections, such as tests of normality and adjustment for multiple comparisons
☐	☒	A full description of the statistical parameters including central tendency (e.g. means) or other basic estimates (e.g. regression coefficient) AND variation (e.g. standard deviation) or associated estimates of uncertainty (e.g. confidence intervals)
☐	☒	For null hypothesis testing, the test statistic (e.g. *F*, *t*, *r*) with confidence intervals, effect sizes, degrees of freedom and *P* value noted<br>*Give P values as exact values whenever suitable.*
☒	☐	For Bayesian analysis, information on the choice of priors and Markov chain Monte Carlo settings
☒	☐	For hierarchical and complex designs, identification of the appropriate level for tests and full reporting of outcomes
☒	☐	Estimates of effect sizes (e.g. Cohen's *d*, Pearson's *r*), indicating how they were calculated

Our web collection on statistics for biologists contains articles on many of the points above.

Software and code

Policy information about availability of computer code

Data collection	Leica M205 FA stereomicroscope was used to capture images of rice florets and lodicules;<br>Zeiss LSM780 confocal laser-scanning microscope was used to capture images in subcellular localization;<br>Biostep CELVIN® S 420 was used to capture images of EMSA and western blot;<br>Roche LightCycler96 real-time PCR system was used for RT-qPCR assay;<br>Promega GloMax® 20/20 Luminometer was used for dual-LUC assay.
Data analysis	ImageJ software was used to measure the length, width and height of the lodicules;<br>RNA-seq data were processed by HTSeq v0.6.1 for gene expression level analysis and DESeq2 R package for differentially experssed genes analysis;<br>DAP-seq data were processed by Bowtie2 v2.3.5.149 for reads alignment and MACS2 v2.1.051 for peaks calling, peaks were identified using the MEME-ChIP online software (https://meme-suite.org/meme/), putative genes associated with peaks were annotated using ChIPseeker v1.32.152;<br>VCFtools v0.1.16 was used to nucleotide diversity (π) and fixation index analyses;<br>GraphPad Prism 8 was used in two-sided unpaired Student’s t-test and one-way analysis of variance (ANOVA);<br>MEGA 7 software was used in sequence alignment and phylogenetic tree construction;<br>Bio-Rad QuantaSoft Analysis Pro software was used to analysis droplet digital PCR data.

For manuscripts utilizing custom algorithms or software that are central to the research but not yet described in published literature, software must be made available to editors and reviewers. We strongly encourage code deposition in a community repository (e.g. GitHub). See the Nature Portfolio guidelines for submitting code & software for further information.

Data

Policy information about availability of data

All manuscripts must include a data availability statement. This statement should provide the following information, where applicable:

- Accession codes, unique identifiers, or web links for publicly available datasets
- A description of any restrictions on data availability
- For clinical datasets or third party data, please ensure that the statement adheres to our policy

We have provided a full data availability statement in the manuscript: All data supporting the findings of this study are available in the main text, supplementary information, and source data files. Source data are provided with this paper. The DAP-seq and RNA-seq data generated in this study have been deposited in the NCBI Sequence Read Archive database under the accession number PRJNA1000954 and PRJNA1000956, respectively.

Research involving human participants, their data, or biological material

Policy information about studies with human participants or human data. See also policy information about sex, gender (identity/presentation), and sexual orientation and race, ethnicity and racism.

Reporting on sex and gender	N/A
Reporting on race, ethnicity, or other socially relevant groupings	N/A
Population characteristics	N/A
Recruitment	N/A
Ethics oversight	N/A

Note that full information on the approval of the study protocol must also be provided in the manuscript.

Field-specific reporting

Please select the one below that is the best fit for your research. If you are not sure, read the appropriate sections before making your selection.

☒ Life sciences ☐ Behavioural & social sciences ☐ Ecological, evolutionary & environmental sciences

For a reference copy of the document with all sections, see nature.com/documents/nr-reporting-summary-flat.pdf

Life sciences study design

All studies must disclose on these points even when the disclosure is negative.

Sample size	The sample sizes and material amount were determined based on experiment traits to allow for confident statistical analyses. All the sample sizes have indicated in figure legends.
Data exclusions	No data were excluded from the analyses.
Replication	All experiments were performed with at least three independently replicates. All attempts at replication were successful.
Randomization	Measurements of samples were performed by randomly selecting plants grown under the same conditions. All samples were allocated randomly into experimental groups.
Blinding	The samples were blindly processed during the experiment.

Reporting for specific materials, systems and methods

We require information from authors about some types of materials, experimental systems and methods used in many studies. Here, indicate whether each material, system or method listed is relevant to your study. If you are not sure if a list item applies to your research, read the appropriate section before selecting a response.

Materials & experimental systems

n/a	Involved in the study
☐	☒ Antibodies
☒	☐ Eukaryotic cell lines
☒	☐ Palaeontology and archaeology
☒	☐ Animals and other organisms
☒	☐ Clinical data
☒	☐ Dual use research of concern
☐	☒ Plants

Methods

n/a	Involved in the study
☒	☐ ChIP-seq
☒	☐ Flow cytometry
☒	☐ MRI-based neuroimaging

Antibodies

Antibodies used

The information of the antibodies used in this study were showed below:
Anti-GFP (#ab290) was purchased from Abcam (Cambridge, UK);
Anti-Actin (#A17910) was purchased from ABclonal (Beijing, China);
Anti-rabbit IgG (#ab205718) was purchased from Abcam (Cambridge, UK).
The antibodies were applied into western blot at the dilution of 1:2000.

Validation

Anti-GFP is a rabbit polyclonal antibody recombinant full length protein corresponding to GFP. https://www.abcam.com/products/primary-antibodies/gfp-antibody-ab290.html
Anti-Actin is a synthetic peptide corresponding to a sequence within amino acids 1-100 of human α-smooth muscle actin (ACTA2). https://abclonal.com.cn/Datasheet/Antibodies/A17910.html
Anti-rabbit IgG used for conjugation reacts with rabbit immunoglobulins of all classes. https://www.abcam.cn/products/secondary-antibodies/goat-rabbit-igg-hl-hrp-ab205718.html

Dual use research of concern

Policy information about dual use research of concern

Hazards

Could the accidental, deliberate or reckless misuse of agents or technologies generated in the work, or the application of information presented in the manuscript, pose a threat to:

No	Yes
☒	☐ Public health
☒	☐ National security
☒	☐ Crops and/or livestock
☒	☐ Ecosystems
☒	☐ Any other significant area

Experiments of concern

Does the work involve any of these experiments of concern:

No	Yes
☒	☐ Demonstrate how to render a vaccine ineffective
☒	☐ Confer resistance to therapeutically useful antibiotics or antiviral agents
☒	☐ Enhance the virulence of a pathogen or render a nonpathogen virulent
☒	☐ Increase transmissibility of a pathogen
☒	☐ Alter the host range of a pathogen
☒	☐ Enable evasion of diagnostic/detection modalities
☒	☐ Enable the weaponization of a biological agent or toxin
☒	☐ Any other potentially harmful combination of experiments and agents
